# Identification of immune-related signature for the prognosis and benefit of immunotherapy in triple-negative breast cancer

**DOI:** 10.3389/fgene.2022.1067254

**Published:** 2022-11-14

**Authors:** Xiaorui Sun, Tiansong Zhang

**Affiliations:** ^1^ School of Basic Medicine Sciences, Fudan University, Shanghai, China; ^2^ Jing’an District Hospital of Traditional Chinese Medicine, Shanghai, China

**Keywords:** triple-negative breast cancer, immune risk score, immune therapy, tumor microenvironment, biomarker

## Abstract

**Background:** There is a lack of biomarkers for predicting the efficacy of immunotherapy in triple-negative breast cancer (TNBC). Hence, we constructed an immune risk score (IRS) model to predict the prognosis of patients with TNBC and evaluate those who are sensitive to immunotherapy.

**Methods:** The ribonucleic acid (RNA) sequencing data, mutation data, and clinical information of TNBC patients were obtained from The Cancer Genome Atlas database. Data of immune-related genes were obtained from the Import and InnateDB databases. The IRS model was constructed using univariate, least absolute shrinkage and selection operator, and multivariate Cox regression analyses, and the predictive ability of the prognostic model was evaluated. Further external validation was performed using the Gene Expression Omnibus (GEO) databases GSE58812 and GSE135565. Data on the clinical characteristics, immune landscape, and immune checkpoint inhibitors used in different risk groups were analyzed. Finally, the drug sensitivity of the patients in the high- and low-risk groups was predicted.

**Results:** The prognostic risk score model comprised six genes: HSPA6, LCN1, ARTN, IL36G, BCL2A1, and CASP12. The area under the curve values at 1 year, 3 years, and 5 years were 0.835, 0.852, and 0.843, respectively, indicating that the model has a good potential for predicting the long-term survival of TNBC patients, which is consistent with the results of the GEO cohort. Compared with the high-risk group, the low-risk group had a better prognosis; more abundant immune-activated cell infiltrates, such as CD8^+^ T cells and CD4 memory-activated T cells, and a higher enrichment of immune-related signaling pathways, such as the cytokine receptor interaction, nucleotide oligomerization domain-like receptor signal pathway, T-cell receptor signal pathway, and B-cell receptor signaling pathway, were observed. In addition, the immune checkpoint encoding genes, such as CD274, CTLA4, PDCD1, and PDCD1LG2 were highly expressed in the low-risk group, which showed that this group was more likely to benefit from immunotherapy.

**Conclusion:** A new IRS gene feature was established to predict the patients’ prognosis and guide immunotherapy. Moreover, it was revealed that several potential therapeutic drugs can be used in high-risk patients who are unresponsive to immunotherapy.

## Introduction

Among female cancers, breast cancer (BC) ranks first in terms of incidence and mortality, seriously threatening women’s lives and health and causing a huge social burden. In 2020, 19.29 million new cancer cases were reported worldwide, and the number of new BC cases increased rapidly to 2.26 million, officially replacing lung cancer (2.2 million) as the most commonly diagnosed cancer worldwide for the first time and accounting for 11.7% of all new cancer cases (Siegel et al., 2020). Triple-negative breast cancer (TNBC) accounts for 10–20.8% of all pathological types of BC (Li et al., 2017) and is described as BC with no expression of estrogen receptors (ERs), progesterone receptors (PRs), and human epidermal growth factor receptor-2 (HER-2) on immunohistochemical examination of cancer tissues. TNBC has a high recurrence rate, a higher risk of metastasis, and is difficult to treat. Compared with other types of BC, TNBC has a poor prognosis and short survival time (Won and Spruck, 2020). Currently, there is no clear or effective treatment for TNBC. While BC treatment has entered the era of molecular typing, and some subtypes have been identified as effective therapeutic targets, the therapeutic targets for TNBC still remain unclear. Chemotherapy is the mainstay of treatment, but only 20% patients respond well to chemotherapy (Waks and Winer, 2019). Therefore, there is an urgent need to identify effective measures for treating TNBC.

Previous clinical studies of a variety of solid tumors have confirmed that immunotherapy can effectively prolong the survival of patients, and this treatment method is expected to provide more treatment options for TNBC patients. Among the subtypes of BC, TNBC has an unclear physical behavior and is difficult to diagnose and treat. The IMpassion130 study brings BC diagnosis and treatment into the era of immunotherapy. TNBC has become the most commonly investigated malignant tumor in immunotherapy studies (Adams et al., 2019). Compared with other subtypes of BC, some features of TNBC may make it more responsive to immunotherapy (Cortes et al., 2020). First, it has been shown that high tumor infiltrating lymphocytes (TILs) patients with levels of immune checkpoint inhibitors (ICIs) had a better prognosis, while patients with TNBC had more TILs. Second, programmed cell death ligand 1 (PD-L1) is highly expressed in the tumor and immune cells of patients with TNBC, which provides a direct target for ICIs and is closely related to the efficacy of anti-programmed death-1 (PD-1) therapy (Buisseret et al., 2017). In addition, TNBC has more non-synonymous mutations that generate tumor-specific neoantigens, thereby activating the antitumor immune response of neoantigen-specific T cells. TNBC has become one of the research hotspots in the field of immunotherapy owing to its high mutation rate, high T-cell infiltration, and high expression of PD-L1. In addition, the interactions between tumor cells, stromal and immune microenvironment played a key role in the response to therapies. These heterogeneity in microenvironment and mechanism in TNBC progression are still poorly understood. Although TNBC shows a higher response to ICIs compared with hormone receptor-positive and HER-2 positive BC, the efficacy of ICIs remains unsatisfactory (Keenan and Tolaney, 2020). To screen patients who are most likely to benefit from monotherapy with ICIs and to develop combination therapies to overcome ICI resistance, specific biomarkers for predicting the efficacy of immunotherapy and immune status for TNBC should be identified.

High-throughput data analysis has assumed an increasingly important role in the clinical management of cancer patients, and the need to identify an increasing number of complex biomarkers has led to the introduction of next-generation sequencing (NGS) technologies in clinical practice (Hussen et al., 2022). RNA-sequencing based on NGS has provided new therapeutic modalities for TNBC by identifying cancer-driving variants and molecular subtypes of TNBC (Hu et al., 2021). Bioinformatic methods can effectively and rapidly resolve the biological complexity of TNBC by integrating large amounts of genomic data. In this study, through the analysis of TNBC genomic data, we identified TNBC immune-related genes, constructed and verified a prognostic model, further discussed the immune landscape of TNBC, and analyzed the drug sensitivity of prognostic targets. Our study aimed to identify the immune-related prognostic markers for TNBC at the molecular and clinical levels, which will facilitate the accurate treatment of TNBC.

## Materials and methods

### Data acquisition and differential gene selection

Part of the RNA sequencing data and mutation data and clinical information of TNBC were obtained from The Cancer Genome Atlas (TCGA) database (https://cancergenome.nih.gov/), including 114 tumor samples and 113 normal samples. The GSE58812 and GSE135565 datasets were downloaded from the Gene Expression Omnibus (GEO) database, where GSE58812 contained 107 TNBC samples and GSE135565 contained 84 TNBC samples (https://www.ncbi.nlm.nih.gov/geo/). The de-batching effect was harmonized and eliminated using combat in R software. Differentially expressed genes (DEGs) were identified between normal and TNBC tissues using the limma R package and visualized as heatmaps using the R software. The |log fold change (FC)| ≥ 2and p value of <0.05 were used as screening criteria. All immune-related genes (IRGs) in the ImmPort and InnateDB databases were merged, and the differentially expressed IRGs were extracted from the intersection of immune genes and all DEGs using the online website Venny 2.1.0 [Venny 1.0 (csic.es)]. Gene annotation enrichment analysis of DEGs was performed using the clusterProfiler R package (Yu et al., 2012), including the Kyoto Encyclopedia of Genes and Genomes (KEGG) (Kanehisa and Goto, 2000) and Gene Ontology (GO) analysis (Gene Ontology, 2008). The GO analysis results were divided into three parts: biological process (BP), cellular component (CC), and molecular function (MF). The GO and KEGG terms with a p value of <0.05 were considered significant, and Metascape was used to visualize the results (Zhou et al., 2019).

### Construction of the immune signature

The prognosis-related genes were identified using the univariate Cox regression method, while the immune-related prognostic features were generated using stepwise least absolute shrinkage and selection operator (LASSO) regression analysis, multivariate Cox proportional hazards models, and the survival package in the R software. The LASSO regression analysis is a regularization and dimensionality reduction method that can be used for biomarker screening in combination with Cox models for survival analysis. A multivariate Cox proportional hazards regression model was used to address the multivariate issues affecting patient’s survival time. Briefly, all seven IRGs significantly associated with prognosis were considered in the LASSO analysis as influencing factors. After incorporating this in the multivariate Cox proportional hazards model, six significant IRGs were retained in multiple calculations. The risk score was calculated using the following formula: Immune Risk Score = (expression value of gene1×Coefgene1) + (expression value of gene2×Coefgene2) +…+ (expression value of geneN×CoefgeneN).

### Verification of the immune signature

Patients in the TCGA cohort were divided into low-risk and high-risk groups according to their median risk score value, and their survival was analyzed using the Kaplan–Meier method and log-rank test (Lanczky and Gyorffy, 2021). The specificity and sensitivity of the risk score for predicting the 1-, 3- and 5-year survival were determined by performing a receiver operating characteristic (ROC) analysis using the Survival ROC R package to estimate the area under curve (AUC) of the predictive model. Finally, the clinical data and scores were combined to perform univariate and multivariate independent prognostic analyses. Principal component analysis (PCA) and t-distributed stochastic neighbor embedding (t-SNE) were used to verify the grouping ability of the model.

### Association between the immune-related prediction model and TNBC immune landscape

The levels of infiltrating immune cells and stromal cells were calculated using the CIBERSORT algorithm (Newman et al., 2015). The single-sample gene set enrichment analysis and ESTIMATE algorithm were used for calculating the immune and matrix enrichment scores to determine the predictive models for the relationship between immune effectors. Potential biological functions relevant to the prediction model were enriched using the gene set enrichment analysis (GSEA) method and gene variation analysis and annotated using the GO databases. In the analysis, p value of <0.05 was considered significantly enriched.

### Immune-related prediction models for predicting the therapeutic benefits

The drug susceptibility data were downloaded from the Genomics of Drug Sensitivity of Cancer website (www.cancerrxgene.org). We investigated the predictive power of the models for evaluating the response of patients to immunotherapy and chemotherapy/targeted therapy drugs. The 50% inhibitory concentration (IC50) values of 138 drugs were extrapolated using the PRROPHIC algorithm and were normally converted. The potential response of patients to immunotherapy was inferred by the correlation between the risk model and ICIs and the expression of immune checkpoint genes in the high- and low-risk groups (*p* < 0.05).

### Protein interaction network and gene expression analysis

The predicted gene–protein interaction networks (https://strin g-db.org/cgi/input.pl) were retrieved from the online STING database, and Cytoscape software version 3.9.1 was used for visualization.

## Results

### Immunocorrelated gene model construction

**FIGURE 1:**
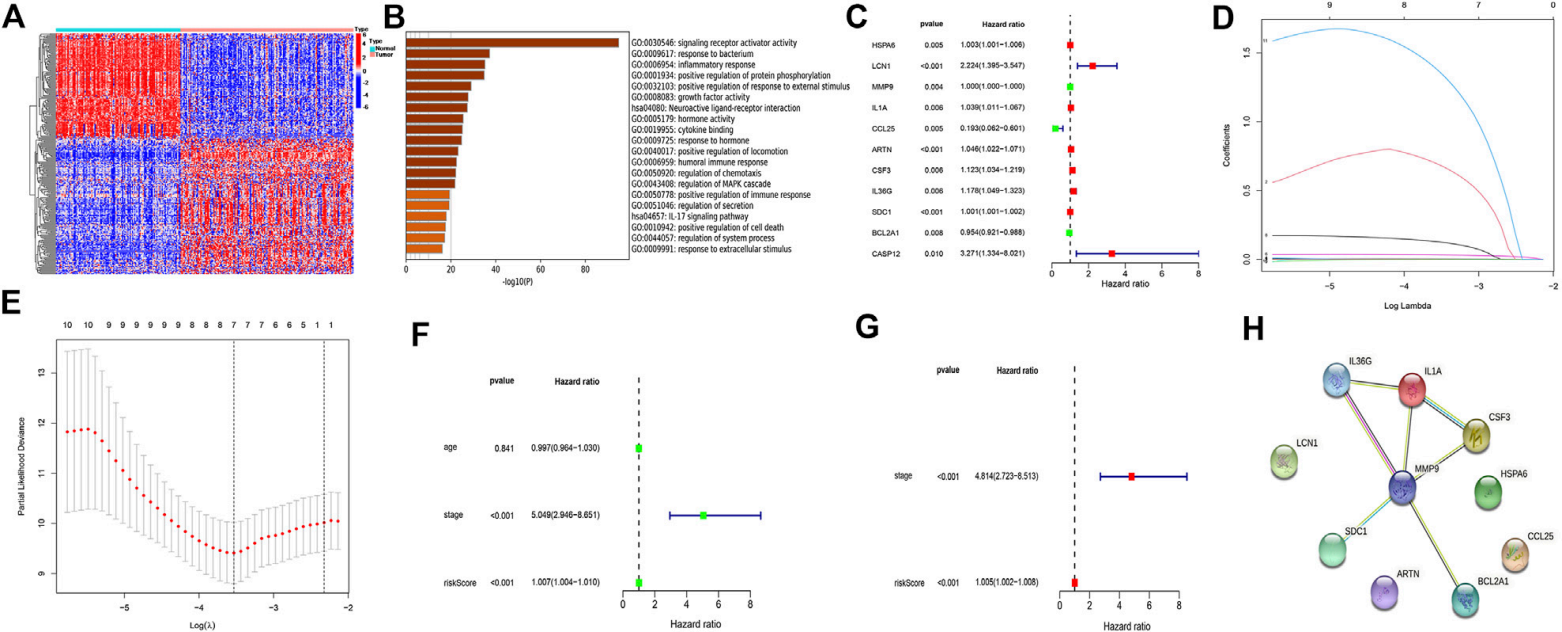
Identifying differential genes and building models. **(A)** Differential genes from TNBC and normal samples. **(B)** Pathway analysis of differential gene enrichment. **(C)** Eleven prognostic-related genes (*p* < 0.05) based on the univariate regression analysis. **(D,E)** Least absolute shrinkage and selection operator Cox regression analysis used to construct a model. **(F)** Univariate regression analysis to assess the model independence. **(G)** Multivariate regression analysis to assess the model independence. **(H)** Protein interplay network.

A total of 290 different genes (*p* < 0.05, |log (FC)|≥2) were identified in normal and TNBC samples, and the heat map accurately reflected the difference between normal and tumor samples (Figure 1A). The p value of <0.05 was used as the critical value for GO functional enrichment analysis and KEGG pathway enrichment analysis. Results of the GO analysis of the molecular function of different genes, which included the inflammatory response, positive regulation of immune response, humoral immune response, and chemotaxis regulation, and the KEGG analysis revealed that the different genes were mainly concentrated in the IL-17 signaling pathway, indicating a strong correlation with inflammatory response (Figure 1B). Univariate regression analysis was performed to identify 11 prognostic-related genes (*p* < 0.01), including 8 tumor-promoting genes (hazard ratio (HR) > 1; heat shock protein family A member 6 (HSPA6), lipocalin-1 (LCN1), interleukin 1 alpha (IL-1α), artemin (ARTN), colony stimulating factor 3, interleukin 36 gamma (IL36G), syndecan 1, and caspase 12 (CASP12) (Figure 1C). LASSO Cox regression analysis yielded the identification of seven immunorelated genes (Figures 1D,E). Six genes (HSPA6, LCN1, ARTN, IL36G, BCL2A1, and CASP12) were identified using a multivariate Cox regression analysis to establish a prognostic risk model (Supplementary Table S1). The model was an independent prognostic factor in the univariate and multivariate regression analyses (Figures 1F,G). A protein–protein interaction network was built for these six genes, of which matrix metalloproteinase-9 was the common node of IL36G and BCL2A1 (Figure 1H). The risk score for each sample was calculated based on six gene expression levels and the corresponding regression coefficients using the following formula: immune risk score (IRS) = (HSPA6 expression value × 0.00491) + (LCN1 expression value × 0.77534) + (ARTN expression value × 0.04608) + (IL36G expression value × 0.17411) + (BCL2A1 expression value × −0.04174) + (CASP12 expression value × 1.45956). The patients were divided into high- and low-risk groups according to their median risk score.

### Validate prognostic risk model

**FIGURE 2:**
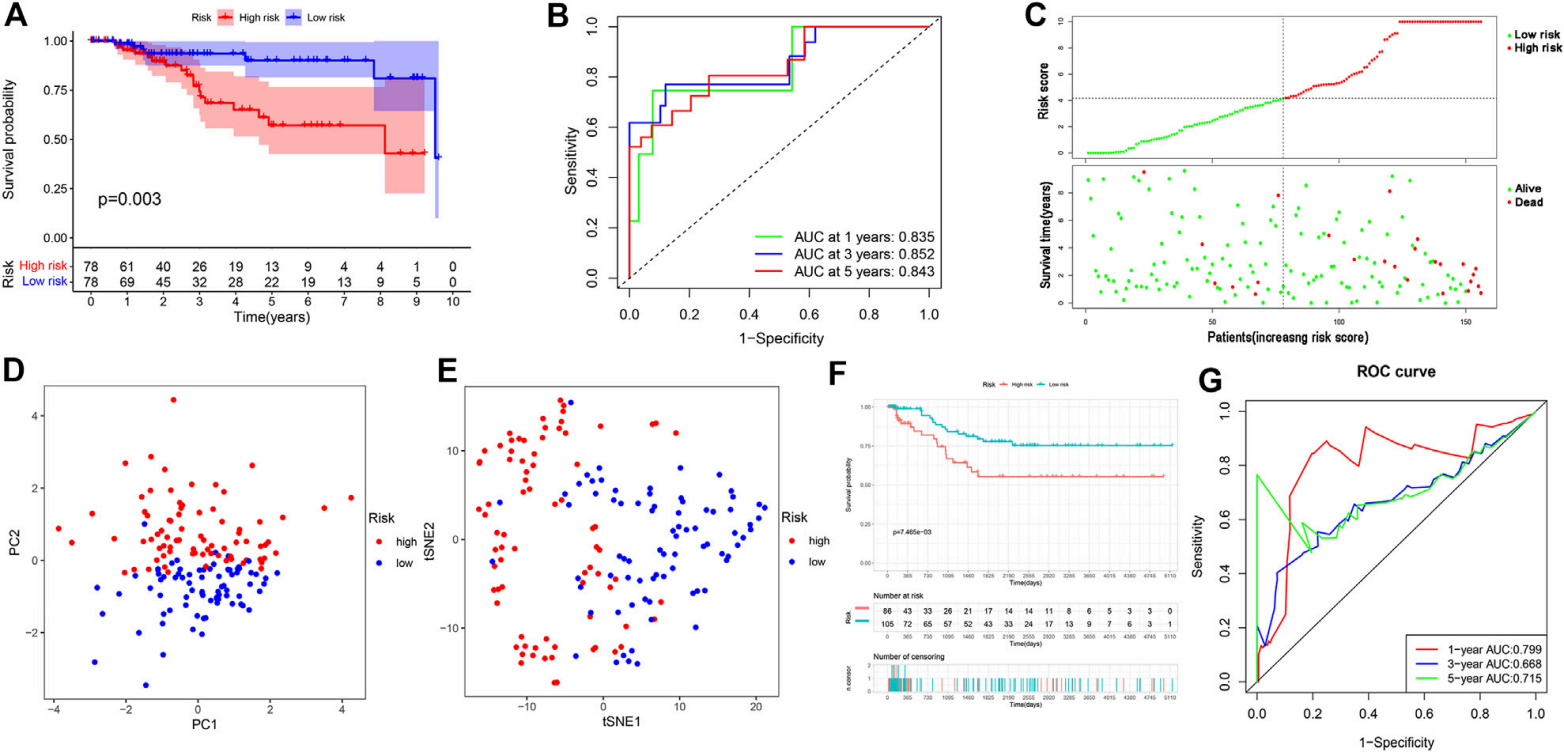
Validation of prognostic risk scoring model. **(A)** Survival analysis between high- and low-risk groups in The Cancer Genome Atlas database. **(B)** The 1-year, 3-year, and 5-year ROC curve values in the TCGA database. **(C)** Survival status of patients with TNBC (high-risk group: right of dotted line; low-risk group: left of dotted line); survival status scatter plots (The state of death: red dots; The state of survival: green dots). **(D,E)** Principal component analysis vs t-distributed stochastic neighbor embedding analysis to verify the grouping effectiveness of the risk scores. **(F)** Survival analysis of the Gene Expression Omnibus (GEO) database. **(G)** ROC curve in the GEO database.

Using the ROC curve and the Kaplan–Meier curve to verify the prognostic value of the model, the overall survival (OS) of the high-risk group was significantly lower than that of the low-risk group (*p* = 0.003) (Figure 2A); the AUC values used to predict the 1-year, 3-year, and 5-year operating curves were 0.835, 0.852, and 0.843 (Figure 2B), respectively. An increase in the risk score was associated with an increased patient mortality (Figure 2C). In addition, PCA and t-SNE analyses showed that the high- and low-risk groups were well distinguished (Figure 2D, Figure 2E). We further verified the dataset in the GEO database, and the OS of the low-risk group was significantly higher than that of the high-risk group (*p* = 7.465e−03) (Figure 2F). The predicted AUC values for the 1-year, 3-year, and 5-year operating curves were 0.799, 0.668, and 0.715, respectively (Figure 2G).

### Immune landscape of high- and low-risk groups

**FIGURE 3:**
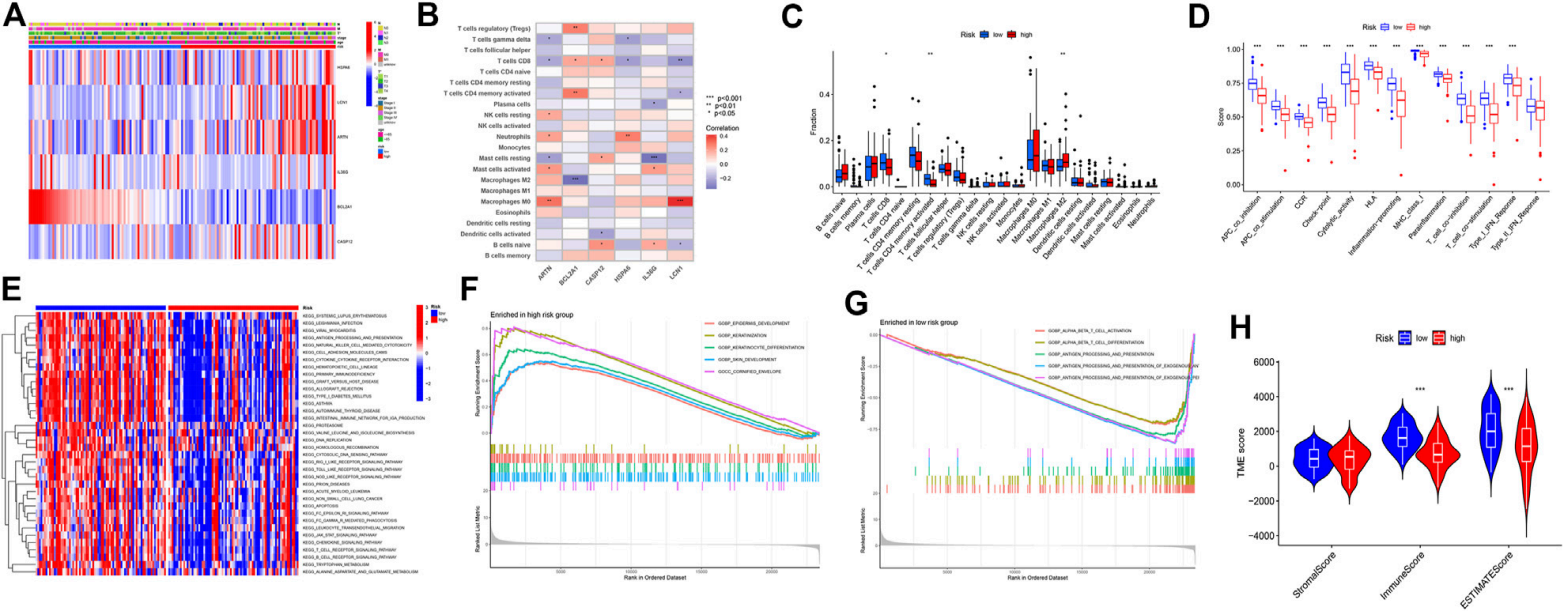
Immune landscape description of the high- and low-risk groups. **(A)** Six-gene expression in the high- and low-risk groups. **(B)** Six-gene expression and immune cell correlation analysis. **(C)** Immunocellular infiltration comparison of high- and low-risk groups. **(D)** Comparison of immune behavior in the high- and low-risk groups. **(E)** Pathway enrichment analysis by GSVA between the high-risk and low-risk groups. **(F)** Gene Ontology (GO) analysis of the high-risk group based on the gene set enrichment analysis (GSEA) results. **(G)** GO analysis of the low-risk group based on the GSEA results. **(H)** stromal, immune, and ESTIMATE scores in the high- and low-risk groups.

The expression levels of HSPA6, LCN1, ARTN, CASP12, and IL36G were higher in the high-risk group, whereas that of BCL2A1 was higher in the low-risk group (Figure 3A). ARTN is positively correlated with the M0 macrophages and negatively correlated with CD8^+^ T cells and gamma delta T cells. BCL2A1 is positively correlated with regulatory T cell (Treg) and CD4^+^ memory T-cell activation. The IL36G levels were negatively correlated with the proportions of resting mast cells. The expression of LCN1 was positively correlated with the activation of M0 macrophages (Figure 3B). The expression levels of CD8^+^ T cells and activated CD4^+^ memory T cells were higher in the low-risk group, while that of the M2 macrophages was higher in the high-risk group (Figure 3C). The cytolytic activity, human leukocyte antigen expression, inflammation-promoting effects, major histocompatibility complex class I (MHC-I) protein expression, T cell co-stimulation, and type I interferon (IFN) response were relatively higher in the low-risk group (Figure 3D). The KEGG pathway enrichment analysis revealed that the cytokine–receptor interaction, primary immunodeficiency, nucleotide oligomerization domain (NOD)-like receptor signaling pathway, B-cell receptor signaling pathway, and T-cell receptor signaling were highly enhanced in the low-risk group (Figure 3E). The biological processes, such as *epidermis* development and cornified envelopes, were significantly enriched in the high-risk group (Figure 3F). The alpha-beta T-cell activation, αβ T-cell differentiation, antigen processing, and presentation were significantly enhanced in the low-risk group (Figure 3G). The immune and ESTIMATE scores were relatively high in the low-risk group (Figure 3H).

### Clinical features of the high- and low-risk groups

**FIGURE 4:**
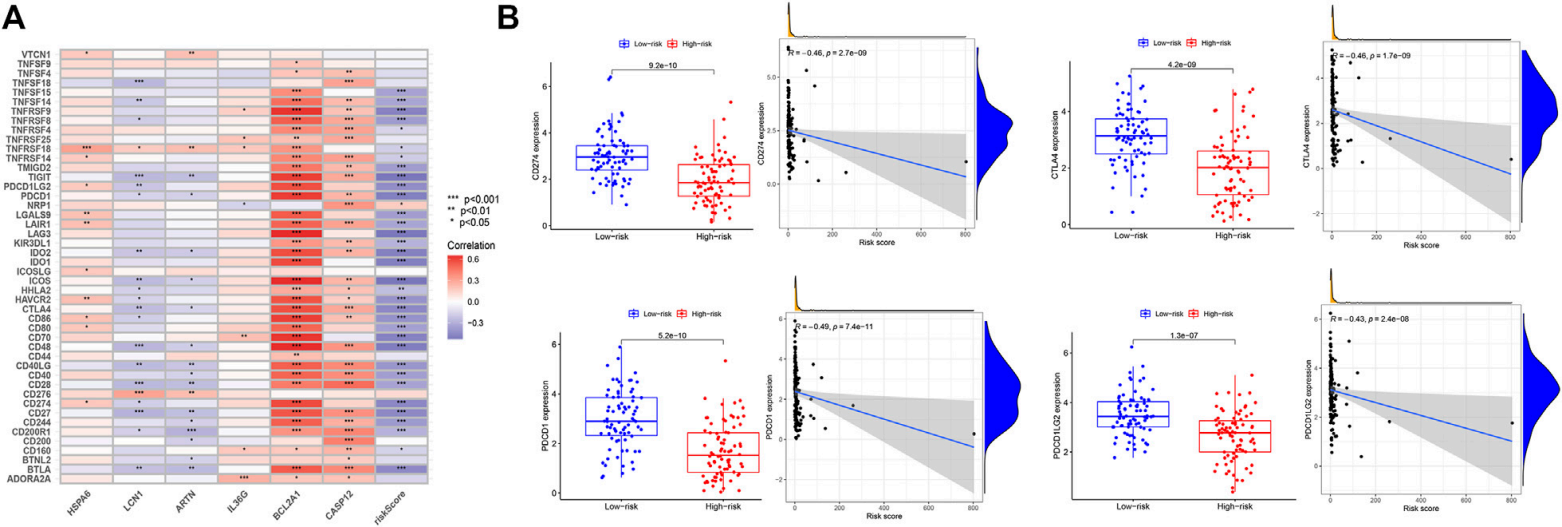
Expression of immune checkpoints in the high- and low-risk groups. **(A)** Correlation analysis between the six genes and immune checkpoints. **(B)** Analysis of the high- and low-risk groups with immune checkpoints CD274, CTLA4, PDCD1, and PDCD1LG2.

CD274 was positively correlated with the expression of HSPA6 and BCL2A1. cytotoxic T-lymphocyte-associated antigen 4 (CTLA4) and PDCD1 were positively correlated with BCL2A1 and CASP12. PDCD1LG2 was positively correlated with the expression of HSPA6 and BCL2A1 (Figure 4A). The expression levels of the immune checkpoint genes CD274, CTLA4, PDCD1, and PDCD1LG2 were significantly high in the low-risk group. That is, the risk score was negatively correlated with the expression of CD274 (R = −0.46, p = 2.7e−09), CTLA4 (R = −0.46, p = 1.7e−09), PDCD1 (R = −0.49, p = 7.4e−11), and PDCD1LG2 (R = −0.43, p = 2.4e−08), respectively (Figure 4B).

### Drug sensitivity analysis

**FIGURE 5:**
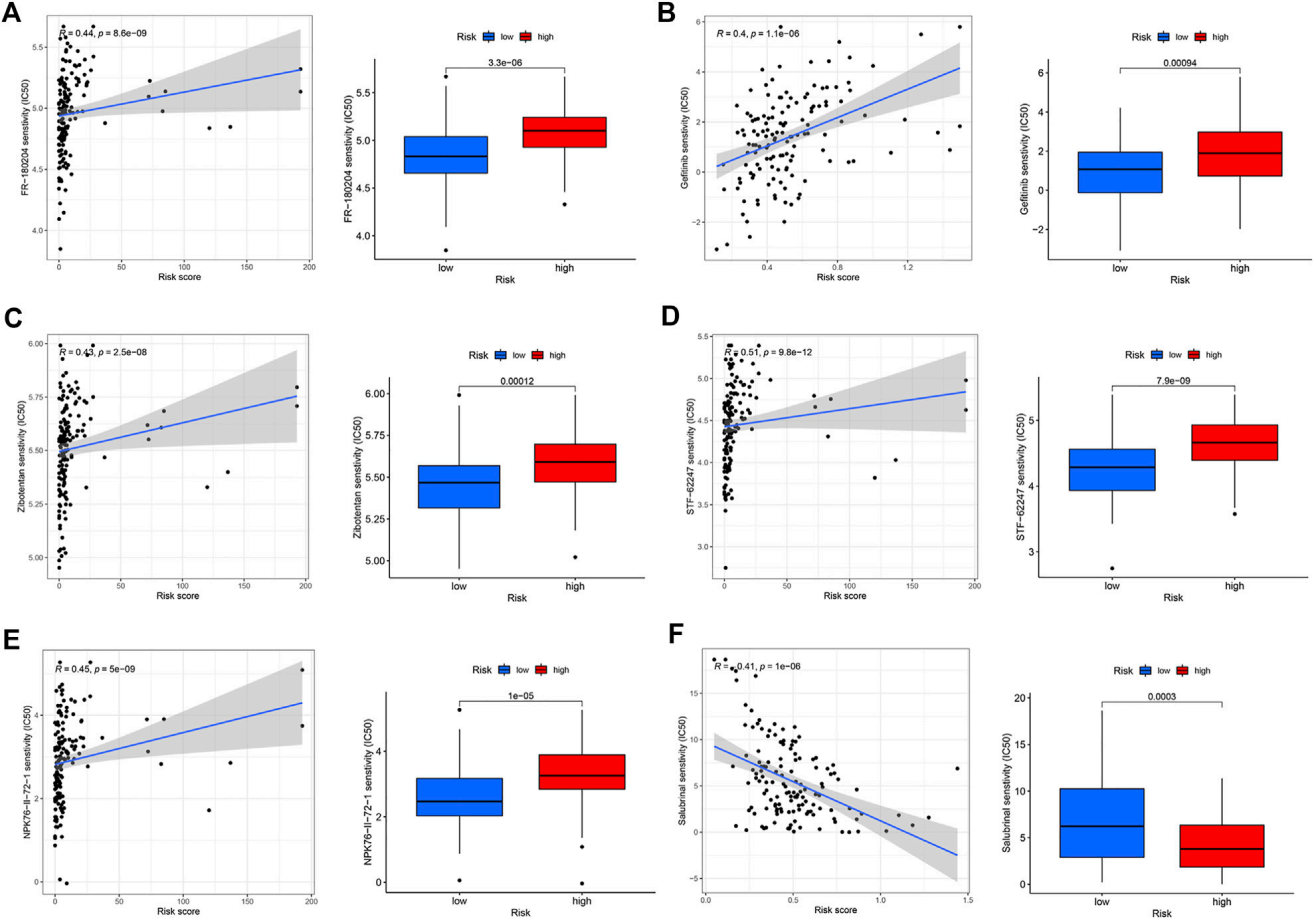
Drug sensitivity analysis of the high- and low-risk groups. **(A)** FR-180204. **(B)** Gefitinib. **(C)** Zibotentan. **(D)** STF-62247. **(E)** Npk76-ii-72-1. **(F)** Salubrinal.

In addition to predicting the patients who are responsive to immunotherapy, we also conducted a drug susceptibility analysis to identify the alternative therapeutic agents for the less-sensitive treatment groups. The low-risk group was sensitive to extracellular signal-regulated kinase (ERK) inhibitors (FR-180204, *p* = 8.6e-09, R = 0.44), epidermal growth factor receptor (EGFR)-tyrosine kinase inhibitors (gefitinib, *p* = 1.1e-06, R = 0.4), endothelin A receptor antagonist (zibotentan, *p* = 2.5e-08, R = 0.43), TGN inhibitors (STF-62247, *p* = 9.8e-12, R = 0.51), and Npk76-ii-72-1 (p = 5e−09, R = 0.45) (Figures 5A–E). The high-risk group was sensitive to the selective eIF2α dephosphorylation inhibitor, salubrinal (p = 1e−06, R = 0.41) (Figure 5F).

## Disscussion

The clinical success of immunotherapies, such as ICIs, has led to the upgradation of traditional tumor treatment strategies. Targeting the immune checkpoints, including the PD-1/PD-L1 axis and CTLA4, can effectively enhance the function of immune cells in order to kill the tumor cells (Desbois et al., 2020). TNBC as a relatively strong immunogenic subtype of BC is more likely to benefit from immunotherapy (Wang et al., 2021). However, the related immune risks have not been fully clarified, and a large number of patients cannot benefit effectively from immunotherapy. Therefore, further identification of this group is of utmost importance. The identification of immune-related gene targets, in-depth understanding of TILs, and analysis of the role of TME in immunotherapy will provide more possibilities for the development of new immunotherapy strategies for TNBC.

We identified the characteristic DEGs from the TNBC and normal breast tissue samples and analyzed their related enrichment pathways. A total of 290 DEGs were enriched in signaling receptor activator activity, inflammatory response, positive regulation of protein phosphorylation, humoral immune response, and other immune-related pathways. This finding indirectly confirmed that TNBC may have higher immunogenicity and a higher probability of benefiting from immunotherapy. Subsequently, the IRS model (HSPA6, LCN1, ARTN, IL36G, BCL2A1, and CASP12) was constructed to predict the immune risk in TNBC patients, which was calculated based on the results of the univariate cox, LASSO, and multivariate cox regression analysis, with the median values used to divide the patients into the high- and low-risk groups. IRS not only showed a good prognostic assessment in the TCGA cohort (AUC at 1 year, 3 years, and 5 years remained >0.83) but also showed moderate prognostic stratification power in other independent validation cohorts, confirming the validity of IRS in describing the prognostic and immune characteristics of TNBC. To further verify the robustness of the model, PCA and t-SNE dimensionality reduction analyses were also performed, except for AUC, which could better judge the clustering effectiveness of the model. Results showed that IRS can better separate the TNBC samples and has good clustering efficiency. Thus, the model provides a potential complementary tool for the clinical immunotyping of TNBC.

In the high IRS group, the expression levels of HSPA6, LCN1, ARTN, CASP12, and IL36G were upregulated, whereas that of BCL2A1 showed the opposite trend. HSPA6, LCN1, ARTN, CASP12, and IL36G were mostly negatively correlated with immune activation-related cells but were positively correlated with M0 macrophages. BCL2A1 was positively correlated with CD4 memory-activated T cells, CD 8 + T cells, and Tregs but was negatively correlated with M2 macrophages. HSPA6 is dispensable for withaferin A-mediated apoptosis/autophagy or inhibition of BC migration (Hahm et al., 2021), its mechanism of action in TNBC is unknown, and other regulatory mechanisms may affect the immune response. LCN1 is overexpressed in BC and associated with poor survival (Zhang et al., 2020). ARTN, a member of the glial cell line-derived neurotrophic ligand family, exerts oncogenic effects on a wide range of solid tumors, including tumor growth, metastasis, and angiogenesis. It can function as a cancer stem cell and transfer factor in BC, and its high expression is often associated with acquired drug resistance among BC patients, residual disease after chemotherapy, relapse, and poor prognosis (Airaksinen and Saarma, 2002; Banerjee et al., 2011; Banerjee et al., 2012; Ding et al., 2014). CASP12, an inflammatory caspase, is closely related to the regulation of inflammatory signaling and plays an important role in apoptosis (Garcia de la Cadena and Massieu, 2016). In cancer pathology, the inflammatory microenvironment and oncogenic mutations often induce chronic inflammation, and CASP12 induction is associated with cancer cell invasion after pre-inflammatory stimulation (Elinav et al., 2013; Chow et al., 2021). The expression levels of IL36G were related to inflammation and were induced by IFN-γ and TNF-α (Ha et al., 2022). The BCL2A1 expression was negatively correlated with IRS. BCL2A1 is a member of the BCL-2 family of anti-apoptotic proteins that confer resistance to anticancer drug therapy (Hiraki et al., 2018).

From the above analysis of the correlation between genes and cells and the analysis of immune cell infiltration, IRS not only effectively indicates tumor progression and prognosis but also more powerfully distinguishes the immune risk of TNBC patients. Individuals with low IRS have a better CD8^+^ T-cell and CD4 memory-activated T-cell infiltration status. In most immunotherapeutic settings, CD8^+^ T cells are major players in eradicating cancer cells. They can recognize tumor-associated antigens, and mediate their cytotoxic effects through the MHC-I molecules (Lorenzo-Herrero et al., 2019). CD4^+^ T cells play an important role in initiating tumor specific CD 8 + T cells and the secondary expansion and memory of CD8^+^ T cells (Janssen et al., 2003). Increased CD4 and CD8 T cell infiltrated in the microenvironment might associated with better survival outcomes. In addition, M2 macrophages are abundantly enriched in populations with high immune risk. M2-type macrophages express Th2 cytokines, such as IL-4, IL-13, and immune complexes, which inhibit the impact of inflammatory factors, most of which play a role in hindering the inflammatory response and tissue repair (Duan et al., 2021). The infiltration state of immune cells and the biological behavior of cells can be well differentiated. In the low-risk group, the immune responses such as antigen-presenting cell co-suppression and co-stimulation, cytolytic activity, inflammation-promoting effects, and co-stimulation of T cells were significantly upregulated in the low IRS group. To a certain extent, more abundant immune-activating cell infiltration and more complex immune responses also indicate immune-stimulatory responses and immunotherapy benefits.

To avoid the biased influence of a single factor, and analyze its immune cells and immune behavior as a whole, the potential pathway mechanism was explored. KEGG pathway enrichment analysis showed that the cytokine–receptor interaction, NOD-like receptor signaling pathway, T-cell receptor signaling pathway, and B-cell receptor signaling pathway were significantly enriched in the low-risk groups. GSEA analysis also demonstrated similar results; the αβ T-cell activation, αβ T-cell differentiation, antigen processing, and presentation effects were more observed in the low-risk group, while embryonic development and other pathways were commonly observed in the high-risk group. These findings implied that the high-risk group might have some stem cell-related properties that were more conducive to the progression of aggressive tumors.

In addition to the analysis at the cellular and molecular levels, in order to better meet the clinical needs, we further analyzed the clinical guidance effect of the 6-gene signature. Most immune checkpoint-related genes have a strong correlation with IRS genes. Moreover, TNBC is more likely to respond to immunotherapy compared with other BC subtypes; with the current immunopharmaceutical therapy, TNBC patients with PD-1 and CTLA4 upregulation are more likely to respond to ICIs (Zhao et al., 2021). Therefore, a correlation analysis of the expression of immune checkpoints between the high and low IRS groups was conducted, and results showed that CD274, CTLA4, PDCD1, and PDCD1LG2 were highly expressed in the low-risk group, which was negatively correlated with the risk score. Therefore, TNBC patients with low IRS were more likely to express immune checkpoint genes to obtain better immunotherapy responses and guide clinical treatment. Considering that not all patients are suitable for immunotherapy, we have provided other drug-sensitive strategies for different subgroups. Our model inferred that an EGFR inhibitor (gefitinib) is also a promising therapeutic target, especially in the low-risk group. Interestingly, A DNA microarray analysis performed by Nielsen et al. showed an overexpression of EGFR in 60% of TNBC samples (Nielsen et al., 2004). In addition, the low-risk group was sensitive to ERK inhibitors (FR-180204), NPK76-II-72-1, TGN inhibitors (STF-62247), and endothelin A receptor antagonists (zibotentan). The high-risk group may be more sensitive to the selective eIF2α dephosphorylation inhibitor, salubrinal.

Although this study provides some evidence on the prognosis, immunophenotyping, and benefits of immunotherapy in TNBC patients, it has some limitations. The inherent limitations of the data from the database are unavoidable; hence, to better reduce the bias caused by this factor, we enrolled samples from different independent cohorts for rigorous validation. In addition, the incomplete clinicopathological information obtained from the database may affect the efficiency of IRS as an independent prognostic factor in the multivariate Cox regression analysis. Hence, future studies should include more clinical samples and laboratory data to better understand the molecular mechanism of the IRS panel, to predict the prognosis and immune subtypes of TNBC, and to verify its real utility in the clinical setting, which will facilitate the development of immune treatment strategy for TNBC.

## Conclusion

In conclusion, a novel IRS gene signature was established to predict the prognosis of TNBC and was validated in TCGA and GEO cohorts. This signature is a potential tool for TNBC survival prediction and immunotherapy guidance. Based on the baseline samples, the IRS gene signature identified patients with a likely high immune infiltrate status who had favorable prognosis and further identified the intercellular biological behaviors and enriched pathways. Patients in the low-risk group had higher immunogenicity and benefited from immunotherapy. This finding may provide more strategies, fresher perspectives, and better directions for personalized diagnosis and treatment of TNBC in the future.

## Data Availability

The datasets presented in this study can be found in online repositories. The names of the repository/repositories and accession number(s) can be found in the article/Supplementary Material.

## Glossary

ARTN: artemin
AUC: area under curve
BCL2A1: B-cell lymphoma 2 related protein A1
BP: biological process
CASP12: caspase 12
CC: cellular component
CTLA4: cytotoxic T-lymphocyte-associated antigen 4
DEGs: differentially expressed genes
EGFR: epidermal growth factor receptor
ERs: estrogen receptors
ERK: extracellular signal-regulated kinase
GEO: Gene Expression Omnibus
GO: Gene Ontology
GSEA: gene set enrichment analysis
HER-2: human epidermal growth factor receptor-2
HR: hazard ratio
HSPA6: heat shock protein family A member 6
IC50: 50% inhibitory concentration
ICIs: immune checkpoint inhibitors
IFN: interferon
IL-1α: interleukin 1 alpha
IL36G: interleukin 36 gamma
IRGs: immune-related genes
IRS: immune risk score
KEGG: Kyoto Encyclopedia of Genes and Genomes
LASSO: least absolute shrinkage and selection operator
LCN1: lipocalin-1
log(FC): log fold change
MF: molecular function
MHC-I: major histocompatibility complex class I
NGS: next-generation sequencing
NOD: nucleotide oligomerization domain
OS: overall survival
PCA: Principal component analysis
PD-1: programmed death-1
PD-L1: programmed cell death ligand 1
PRs: progesterone receptors
RNA: ribonucleic acid
ROC: receiver operating characteristic
TCGA: The Cancer Genome Atlas
TILs: tumor infiltrating lymphocytes
TNBC: triple-negative breast cancer
Treg: regulatory T cell
t-SNE: t-distributed stochastic neighbor embedding
