# Supplementary material for: Identification of immune-related signature for the prognosis and benefit of immunotherapy in triple-negative breast cancer
Source: Front Genet. 2022 Nov 14;13:1067254. doi: 10.3389/fgene.2022.1067254 (PMC9701826; doi:10.3389/fgene.2022.1067254)
Supplement: Supplementary file 1 [file Table1.DOCX]

| Table S1. Multivariate Cox regression analysis of the six genes. | | | | | |
| --- | --- | --- | --- | --- | --- |
| ID | Coef | HR | HR.95L | HR.95H | P value |
| HSPA6 | 0.004915 | 1.004927 | 1.002215 | 1.007646 | 0.000364 |
| LCN1 | 0.77534 | 2.171331 | 1.281686 | 3.678498 | 0.003943 |
| ARTN | 0.046083 | 1.047161 | 1.020457 | 1.074565 | 0.000472 |
| IL36G | 0.174115 | 1.190193 | 1.068447 | 1.325811 | 0.001564 |
| BCL2A1 | -0.04174 | 0.959115 | 0.922439 | 0.997249 | 0.035866 |
| CASP12 | 1.459566 | 4.304089 | 1.607431 | 11.52471 | 0.003679 |
